# Topical Effect of a Medically Prescribed Pediatric Antibiotic on Dental Biofilm: A Cross-Over, *In Situ* Study

**DOI:** 10.1371/journal.pone.0055558

**Published:** 2013-01-31

**Authors:** Viviane Santos da Silva Pierro, Dennis de Carvalho Ferreira, Hugo Emiliano de Jesus, Alexandre Soares Rosado, Ronir Raggio Luiz, Kátia Regina Netto dos Santos, Lucianne Cople Maia

**Affiliations:** 1 Department of Pediatric Dentistry and Orthodontics, School of Dentistry, Universidade Federal do Rio de Janeiro (UFRJ), Ilha do Fundão, Rio de Janeiro, Brazil; 2 Institute of Microbiology Paulo de Goés, Universidade Federal do Rio de Janeiro (UFRJ), Ilha do Fundão, Rio de Janeiro, Brazil; 3 Institute of Studies of Public Health (IESC), Universidade Federal do Rio de Janeiro (UFRJ), Ilha do Fundão, Rio de Janeiro, Brazil; 4 Department of Pediatric Dentistry and Orthodontics, School of Dentistry, Universidade Federal do Rio de Janeiro (UFRJ), Ilha do Fundão, Rio de Janeiro, Brazil; Institut Pasteur, URA CNRS 2172, France

## Abstract

**Objective:**

This study aimed to investigate the possible topical effect of a broad-spectrum antibiotic on dental biofilm formed *in situ* in the absence or presence of sucrose.

**Methods:**

A crossover study was conducted in three phases of 14 days each, during which 11 volunteers wore palatal devices containing 6 enamel blocks covered with meshes to allow biofilm formation. Dental blocks were extraorally submitted to a 20% sucrose solution at three different frequencies of exposure (0, 3 and 8 times/day), and to a suspension of amoxicillin/clavulanate potassium (A/CP) or a placebo (P) suspension at an 8-hour time interval application regimen. On the 14^th^ day of each phase, biofilms were collected for microbiological (conventional culture) and molecular (Denaturing Gradient Gel Electrophoresis – DGGE) analyses.

**Results:**

In the absence of sucrose exposure (SE) and at the 3-time daily frequency, dental biofilms treated with A/CP showed lower total biofilm weight and lower counts of total microbiota than the ones treated with P (*p*>0.05). A/CP presented higher counts of *Candida* spp. when compared with P in the presence of SE, especially at the 8-time daily frequency (*p*<0.05). Considering the DGGE analysis, the mean number of bands was higher for P (*p*>0.05), regardless of SE. However, DGGE profiles demonstrated large interindividual variability.

**Conclusion:**

Both conventional culture and DGGE have demonstrated some differences on total microbiota of dental biofilms when exposed to the A/CP or P suspensions, mainly in the absence of sucrose, which suggests a possible topical effect of the sugar-free A/CP suspension on dental biofilm.

## Introduction

Dental caries is an infectious disease caused by oral acidophilic bacteria which metabolize fermentable sugars. It is viewed as a consequence of an imbalance in the resident microflora due to frequent conditions of low pH in plaque biofilms, for example, as a result of a sugared diet or a reduction in saliva flow [1]. Therefore, considering the role of oral bacteria in dental caries, frequent oral intakes of medically prescribed antibiotic suspensions could influence caries establishment because of their possible additional topical effect on dental biofilms.

Some clinical studies tried to elucidate the role of antibiotic suspensions on children's oral health status [2]–[11]. Although most of them have been conducted with sick children taking long-term antimicrobial medication compared with their healthy siblings or students from near communities [2]–[7], conflicting results pointed out either a protective effect [2]–[6], [10]–[11] or a contributing effect [7], [9] of antibiotics on dental caries.

Many liquid pharmaceutical preparations (including antibiotics) for children are made palatable by the addition of sucrose, glucose, or fructose as sweeteners. These fermentable carbohydrates in thick liquid preparations may contribute significantly to the dental caries potential in young patients [12]. Furthermore, preoccupation with the main medical problem often results in neglect of oral health, and sugars are frequently offered to patients as a demonstration of comfort or as a necessary source of energy [13]–[14]. With the increasing formulation of sugar-free medicines [15]–[16], the frequency of dietary sugar consumption can be considered of further importance for sick children receiving recurrent and/or prolonged oral medication.

The relationship between medically prescribed antibiotics and dental caries is mainly based on the possible antibacterial effect of these medications on dental biofilm, which could be considered an additional effect of this therapeutic class on oral health status. Considering that amoxicillin and its combined formulation with clavulanate potassium have been pointed out as the most commonly prescribed antibiotics for hospitalized children [17] and for preschool children [18] to treat a variety of infections usually through oral administration, the present study aimed at evaluating, *in situ*, the possible topical effect of a sugar-free antibiotic suspension containing amoxicillin/clavulanate potassium on dental biofilm.

## Methods

### Ethics Statement

This *in situ* study was approved by the Research and Ethics Committee of IESC/UFRJ (protocol No. 03/2010), and was performed with undergraduate and graduate dental students who signed an informed consent for participation. All procedures were conducted in accordance with the Declaration of Helsinki.

### Experimental Design

**Figure 1:**
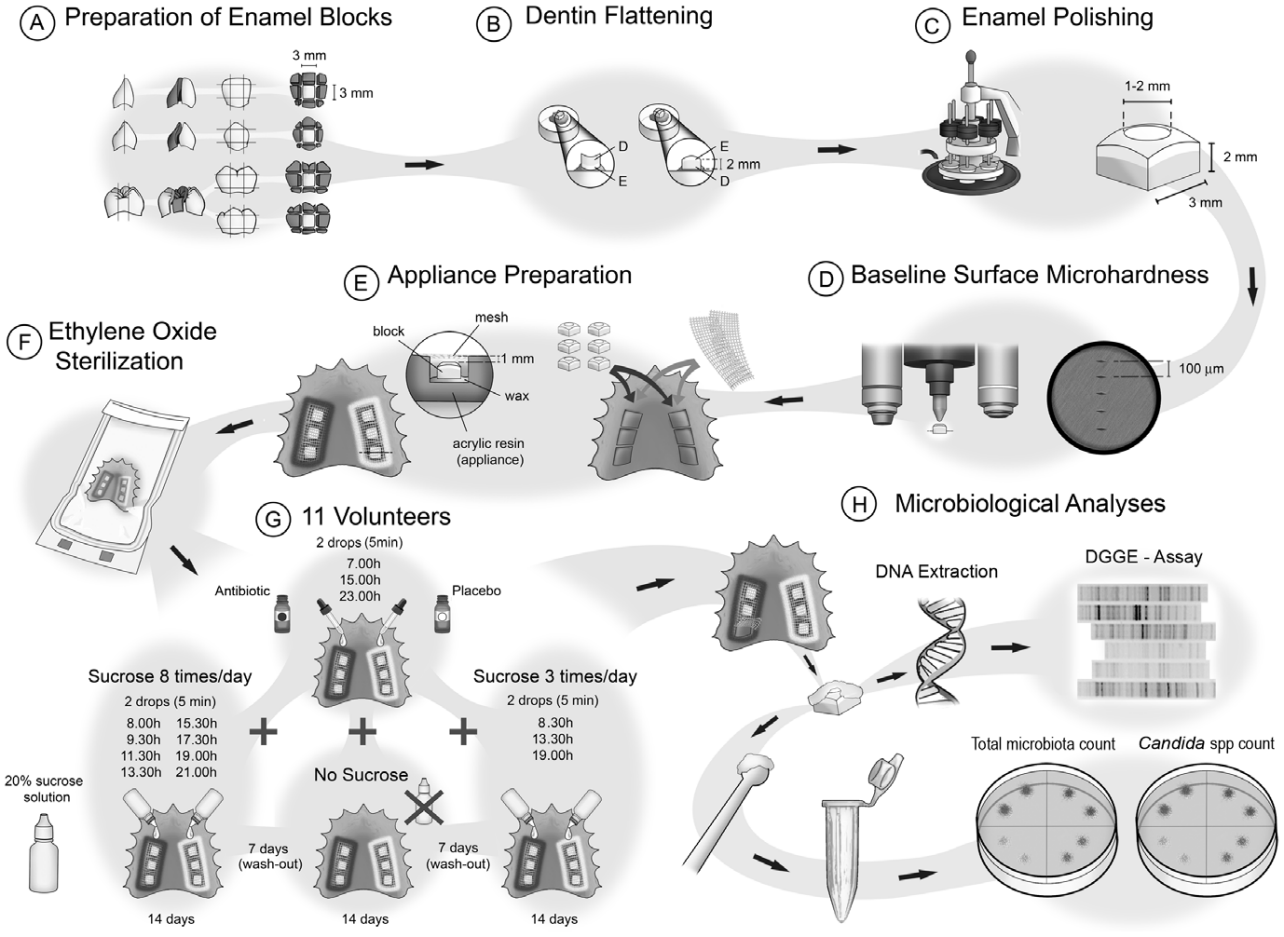
Schematic illustration of the experimental design.

The study had a crossover and “split-mouth” design, performed in three experimental phases of 14 days each; with a washout period of 7 days between each phase (Figure 1).

Eleven volunteers (3 males and 8 females, aged 19–31 years) met the inclusion criteria proposed by Zero [19] for *in situ* studies and did not violate the main exclusion criterion, which was the use of antibiotics or any other antimicrobial substances 60 days prior to the study and throughout all experimental phases. Each volunteer wore acrylic intraoral palatal devices containing 2 sets of 3 blocks (3×3×2 mm) of polished sound human primary dental enamel covered by a plastic mesh [20]. Both sets of dental enamel blocks were extraorally submitted to 20% sucrose solution (two drops onto each set) at three different daily frequencies of exposure (no exposure – 0 times/day; exposure – 3 times/day and 8 times/day), which were randomly assigned to each experimental phase undertaken by each volunteer. Additionally, during all the phases, volunteers were instructed to remove the palatal devices and drip two drops of an sugar-free antibiotic suspension containing 250 mg of amoxicillin and 62.50 mg of clavulanate potassium/5 mL onto one set of enamel blocks, and two drops of a placebo suspension onto the other set of blocks, following an 8-hour time interval application regimen. All volunteers were blind to this treatment regimen [21]–[25]. Furthermore, excess fluid was removed with gauze and the device was replaced in the mouth after 5 minutes [24]–[25]. The use of two treatments (split-mouth) in the same intraoral device was supported by the absence of a crossover effect in previous studies [21]–[26]. All intraoral devices were sterilized with ethylene oxide before the beginning of each experimental phase.

The volunteers received instructions to wear the devices all the time, including at night, but to remove them during meals, drinking and oral hygiene. All volunteers were advised to keep their usual dietary pattern during all the experimental phases.

### Dental Biofilm Collection

On the 14th day of each experimental phase, plastic meshes were cut with sterile surgical blades (No. 11) and dental biofilms formed on the 2 opposite sets of 3 enamel blocks were separately collected with sterilized plastic curettes for microbiological analyses.

The collected biofilms were weighed in pre-weighed sterile microcentrifuge tubes and approximately 1.0–3.0 mg (wet weigh) were transferred to other sterile microcentrifuge tubes containing 5 glass beads (2 mm in diameter) to be suspended in 1 mL of 0.85% NaCl solution. The biofilms were vortexed for 30 s and ten-fold serial dilutions in sterile saline were inoculated in duplicate by the drop-countig technique [27] on the following culture media: blood agar (Plast Labor, Brazil), for total microbiota; and CHROMagar Candida (CHROMagar, France), for *Candida* spp. CHROMagar plates were incubated for 48 h at 37°C in aerobiosis, while blood agar plates were incubated in microaerophilic condition (candle jar) at 37°C for 24 h. The colony-forming units (CFU) were counted and the results expressed in number of CFU/mg dental biofilm (wet weight) and converted to log_10_ for the statistical analyses.

To proceed with bacterial molecular analysis, an aliquot of 300 µL from the above mentioned original suspensions was transferred to microcentrifuge tubes containing 1.0 mL of TE buffer (10 mM Tris- and 1 mM EDTA [pH 7.8]), which were agitated and transported on ice to a microbiology laboratory at the Dept. of Microbiology (UFRJ).

### DNA extraction

The 1.3 mL samples were vortexed for 30 sec. Then, the microbial suspension was washed three times with 100 µL of sterilized Milli-Q water and bacteria were pelleted from the suspension by centrifugation at 2,500×g. The pellets were resuspended in 100 µL of Milli-Q water and bacterial DNA was extracted using QIAamp DNA Mini Kit (Qiagen, USA) according to the manufacturer's instructions. All DNA was stored at −20°C before further analysis.

### PCR assay

An aliquot of 5 µL of the extracted DNA was used for PCR amplification and the resultant products were further submitted to Denaturing Gradient Gel Electrophoresis (DGGE).

A 16S rRNA gene fragment corresponding to nucleotide positions 968–1401 (*Escherichia coli* numbering) was amplified from DNA extracts of dental biofilms using the following universal bacterial primers: 968f (5′-AAC GCG AAG AAC CTT AC- 3′) containing a 40 bp GC clamp (5′-CGC CCG CCG CGC GCG GCG GGC GGG GCG GGG GCA CGG GGG G-3′) added to its 5′- end, which makes it suitable for DGGE, and 1401r (5′-CGG TGT GTA CAA GAC CC-3′) [28].

The PCR mixture comprised 5 µL of DNA from clinical samples, 10 pmol universal primers, 5 µL PCR buffer 10×, 2.5 mM MgCl_2_, 2.5 U Taq DNA polymerase, 10 pmol concentration of each deoxynucleoside triphosphate and sterilized Milli-Q water to a final volume of 50 µL. Negative controls consisting of sterilized Milli-Q water instead of sample were included with each batch of samples analyzed. PCR amplification was performed in a DNA thermocycler (Eppendorf AG, Hamburg, Germany). The temperature profile included an initial denaturation step at 94°C for 4 min, followed by 35 cycles of a denaturation step at 94°C for 1 min, a primer annealing step at 55°C for 1 min, an extension step at 72°C for 2 min and a final step of 72°C for 10 min. Before the DGGE analysis, the presence of PCR products was checked by electrophoresis in a 1.2% agarose gel followed by ethidium bromide (2 µg/mL) staining. Gels were visualized under ultraviolet illumination and a 100-bp DNA ladder (New England BioLabs, USA) was used as molecular size marker.

### DGGE assay

DGGE of PCR products generated with the 968f-GC/1401r primer set was performed using the Dcode Universal Mutation Detection System (Bio-Rad Dcode, USA) at 75 V and 60°C for 16 h in 1× TAE buffer (20 mmol Tris/acetate, pH 7.4; 10 mmol sodium acetate; 0.5 mmol disodium EDTA). The PCR products from biofilm samples (30 µL) were loaded on 6% (w/v) polyacrylamide gels containing a linear gradient ranging from 40% to 70% denaturant (100% denaturing solution contains 7 mol urea and 40%, v/v, formamide) and increasing in the direction of electrophoresis. All gels were loaded with DNA markers in the first and last lanes surrounding the lanes with samples to allow gel standardization according to the manufacturer's instructions. After electrophoresis, the DGGE gels were stained with SYBR green I (Molecular Probes, Netherlands) diluted in 20 µL of 1× TBE buffer for 40 min and visualized using a Storm PhosphorImager (Amersham Biosciences, Sweden).

### Analysis of Microbial Profiles by DGGE

Individual lanes of the DGGE gel images were straightened and aligned with GelCompar software (GelCompar II Software, version 5.10, Applied Maths, Belgium). Dendrograms for diverse comparisons of DGGE banding patterns were constructed and analyzed by the BioNumerics software version 6.0 (Applied Maths, Belgium).

### Statistical Analysis

Volunteers were considered as statistical blocks and a factorial 2×3 was considered for the statistical analysis of all variables. The factors under evaluation were: treatment at 2 levels (amoxicillin/clavulanate potassium or placebo) and daily frequency of sucrose exposure at 3 levels (no exposure – 0 times/day; exposure – 3 times/day and 8 times/day). All statistical analyses were performed with the SPSS Software version 17.0 (Statistical Package for Social Sciences, SPSS Inc., USA) and the significance limit was set at 5%.

## Results

Table 1 shows the results of total biofilm weight together with total microbiota and *Candida* spp. counts. The amount of biofilm formed on the dental blocks with the placebo treatment was greater than that of amoxicillin/clavulanate potassium treatment (*p*>0.05), except for the high frequency of sucrose exposure (8 times per day). Regardless of treatment group, sucrose exposure was associated with a greater amount of biofilm accumulation (*p*<0.05 only for the antibiotic group).

**Table 1:** Total biofilm weight and microbiological colony count of total microbiota and *Candida* spp. according to treatments (amoxicillin/clavulanate potassium or placebo) and daily frequency of sucrose exposure (0 – no exposure; exposure – 3 or 8 times/day).

Treatment	Sucrose Exposure (times/day)	Total Biofilm Weight (wet weight in mg)	*Candida* spp CFU×10^6^/mg biofilm	Total Microbiota CFU×10^6^/mg biofilm
Amoxicillin/Clavulanate Potassium	0	3.00 (0.60–16.10)^a,A^	0.42 (0.002–7.81)^a,A^	3.86 (0.02–53.13)^a,A^
	3	5.30 (1.00–66.00)^a,B^	0.51 (0.00004–6.50)^a,A^	1.39 (0.36–27.08)^a,A^
	8	14.50 (5.70–55.60)^a,B^	1.58 (0.06–9.79)^a,A^	3.41 (0.36–46.74)^a,A^
Placebo	0	5.90 (0.90–32.10)^a,A^	0.67 (0.006–2.38)^a,A^	7.50 (0.02–50.00)^a,A^
	3	7.50 (0.70–76.20)^a,A^	0.26 (0.0002–5.00)^a,A^	3.19 (0.06–24.29)^a,A^
	8	9.40 (1.00–83.80)^a,A^	0.86 (0.0003–4.06)^b,A^	2.71 (0.04–72.50)^a,A^

Results are expressed as median (minimum – maximum values) – *n* = 11.

CFU: colony-forming units.

Distinct lower-case superscript letters indicate statistical significance for the comparison between treatments within each frequency of sucrose exposure (*p*<0.05; Wilcoxon Signed-Rank test).

Distinct capital superscript letters indicate statistical significance for the comparison among different frequencies of sucrose exposure within each treatment (*p*<0.05 – Friedman test; *p*<0.017 – *Post-hoc* analysis with Wilcoxon Signed-Rank Tests conducted with a Bonferroni correction).

Total microbiota count was higher in the biofilm of the placebo group in the absence of sucrose and at a 3-time daily frequency of sucrose exposure (*p*>0.05). In the presence of sucrose, populations of *Candida* spp. were higher in the antibiotic group when compared with placebo group, especially at the 8-time daily frequency (*p*<0.05).

Based on the DGGE profile analysis, a total of 54 distinct bands were detected. The mean numbers of bands, indicative of the number of bacterial species, were higher in samples from placebo group (*p*>0.05), regardless of the daily frequency of sucrose exposure. Also, considering the different conditions of sucrose exposure for each treatment (placebo or antibiotic), the mean numbers of bands was higher in the absence of sucrose exposure than in its presence at a high daily frequency (*p*>0.05) for both treatments. The mean ranks of detected bands, indicative of the position of the bands in the gels, were lower in samples from placebo when compared with those from amoxicillin/clavulanate potassium (*p*>0.05) for the three different frequencies of sucrose exposure. These data are shown in Table 2.

**Table 2:** Mean number and mean rank of bands according to treatments (amoxicillin/clavulanate potassium or placebo) and daily frequency of sucrose exposure (0 – no exposure; exposure – 3 or 8 times/day).

Treatment	Sucrose Exposure (times/day)	Total no. of distinct bands on DGGE	Mean no. of bands ± SD	Mean rank of bands ± SD
**Amoxicillin/Clavulanate Potassium**	0	41	9.91±3.24	31.79±4.30
	3	47	10.36±4.57	30.77±4.87
	8	39	8.91±2.39	30.63±3.67
**Placebo**	0	42	10.27±3.07	30.90±6.36
	3	47	11.36±3.59	29.42±4.90
	8	41	9.27±2.87	29.45±3.76

SD: standard deviation; *n* = 11.

With regard to the mean number and the mean rank of bands, no statistical significance was found either for different treatments (*p*>0.05; Paired T-test) or for different frequencies of sucrose exposure (*p*>0.05; Repeated Measures ANOVA test).

Note: Although 54 distinct bands have been detected in the DGGE profile analyses, none of the volunteers presented all of them in their profile.

**Figure 2:**
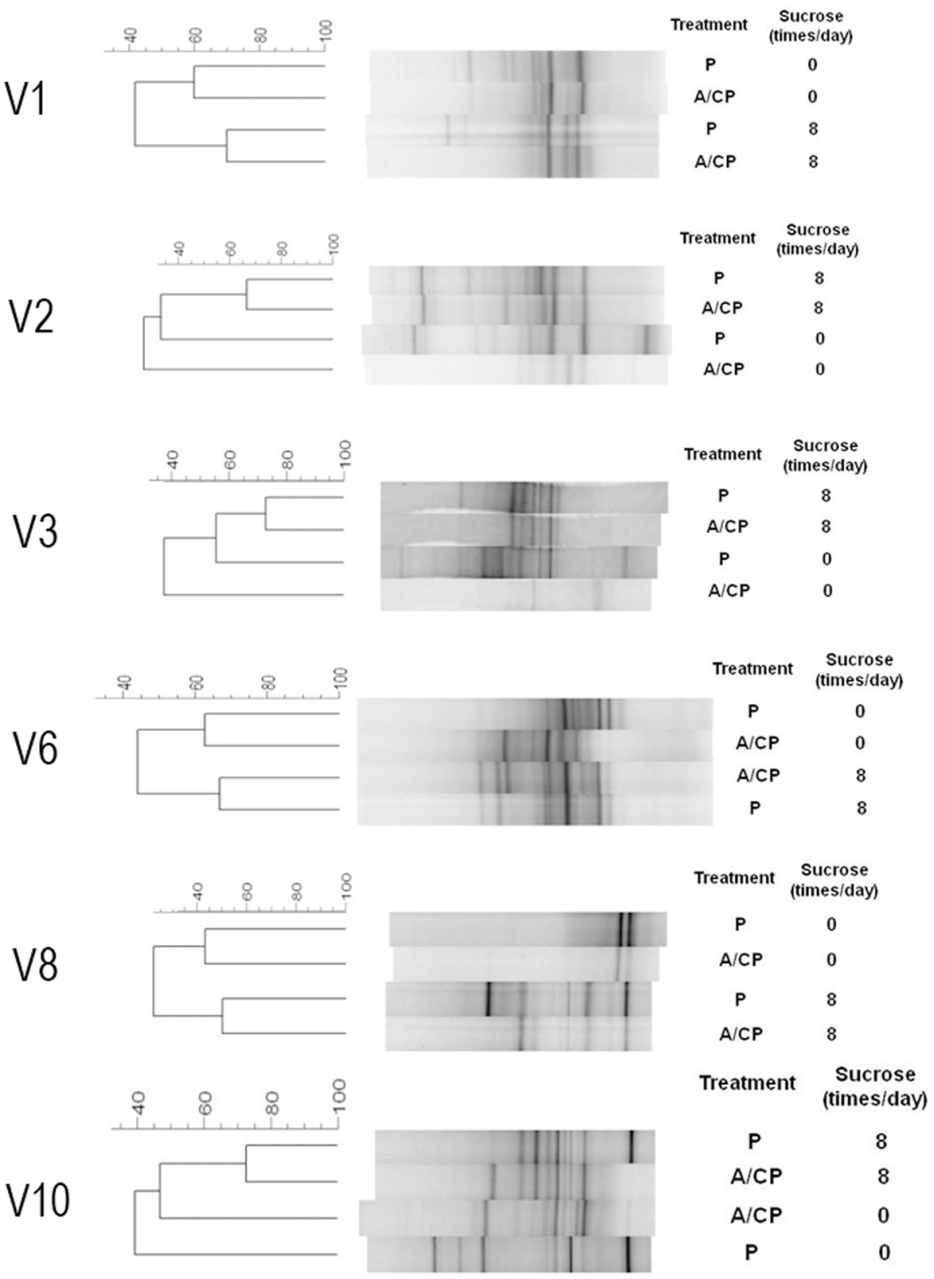
DGGE profiles of PCR-amplified bacterial 16S rDNA gene fragments from biofilm samples of six volunteers. All samples were treated with Placebo (P) or Amoxicillin/Clavulanate Potassium (A/CP) without additional sucrose exposure (0) and at an 8-time daily frequency of 20% sucrose exposure. Comparisons were made for the same volunteer.

DGGE profiles showed a large interindividual variability and it was not possible to determine any specific pattern associated with placebo or amoxicillin/clavulanate potassium for any of the sucrose's frequencies. However, when analyzing the DGGE profile of each volunteer according to treatments and daily frequency of sucrose exposure, an influence of sucrose on the effect of amoxicillin/clavulanate potassium on microbial distribution was verified in six cases (volunteers 1, 2, 3, 6, 8 and 10). In those cases, the patterns of bacterial distribution for placebo and amoxicillin/clavulanate potassium showed more similarity between each other at the 8-time daily frequency of sucrose exposure than in the absence of sucrose (Figure 2). The 3-time daily frequency of sucrose exposure could not be included in this comparison because it showed inconsistent similarity patterns.

## Discussion

The possible additional effect of medically prescribed oral antibiotics on dental biofilm could be mainly due to antibiotic's salivary concentrations after ingestion [29], but also due to a possible topical effect of pediatric liquid formulations on oral biofilm.

Several *in situ* studies have evaluated microbiological characteristics of dental biofilm accumulated on intraoral palatal appliances after treatment with different substances, such as sucrose, fructose and/or glucose [20], [24], [26], [30]; starch [25], [31]; infant formulas [21]; and fluoridated products [22]–[23]. All of them studied the microbial changes of dental biofilms mainly by counting the colony-forming units of known caries-related microorganisms (*e.g.*, *S. mutans*, *Lactobacillus*) and/or total microbiota on culture plates. Nevertheless, some studies have suggested that bacterial species other than *S. mutans* should play important roles in the initial caries process [32] and that over 60% of the oral bacterial flora is represented by not-yet-cultivated phylotypes [33]. For these reasons, the target microbial population evaluated in the present study was the total microbiota (not only the known caries-related microorganisms) of dental biofilm by using conventional culture analysis and a molecular assay as well.

The role of antibiotics on oral microbiota is still a controversial issue. Some clinical studies have shown a relationship between antibiotic usage for medical purposes and a decline in caries-related bacteria and dental caries [2]–[6], [10]–[11]. Conversely, two clinical studies [7], [9] showed a positive relationship between dental caries in primary teeth and oral antibiotic intake. Mariri *et al* [8], however, did not show either a contributing or a protective effect of medically administered antibiotics on dental caries experience in primary dentition. That finding was pointed out by the authors as probably due to the stronger relationships between diet, fluoride and dental caries, which may have overwhelmed the effects of antibiotics. The authors also discussed a possible role of the substantial amounts of sugar present in some antibiotic suspensions counter-balancing the antibiotic effects [8].

The clinical studies investigating the association between medicines and dental caries mentioned above have shown many advantages when compared with experimental ones. However, the study of multifactorial diseases like dental caries may be compromised by confounding factors if not adequately controlled in clinical situations. Therefore, a good alternative to study the role of substances on dental caries are the *in situ* caries models, which involve the use of appliances or other devices that create defined conditions in the human mouth to simulate the process of dental caries [34].

In the present study, we chose an antibiotic suspension containing amoxicillin/clavulanate potassium not only because it is a largely prescribed antibiotic for children, but also because it is available as a sugar-free formulation. Nevertheless, sugars are frequently offered to sick children [13]–[14], and for this reason, different frequencies of sucrose exposure (0 – no exposure; exposure – 3 or 8 times/day) were used in this study to simulate situations of none, low and high cariogenic challenges. Results showed that although the total biofilm weight increased significantly in presence of sucrose exposure, the number of colony-forming units for total microbiota was lower at 3 and 8 times of sucrose exposure per day when compared with the absence of sucrose exposure, for both placebo and antibiotic treatments. These findings could be explained by the feast-or-famine lifestyle lived by bacteria in the oral cavity, where only some of them are able to survive long periods of carbohydrate starvation and sudden exposure to an excess amount of sugar. Therefore, only the bacteria able to develop physiologic and genetic mechanisms to regulate sugar metabolism according to source and availability will survive in the dental biofilms [35]. Furthermore, despite a lower number of total microbiota counts in the presence of sucrose for both treatments, total biofilm weight was significantly higher when sucrose was added to this *in situ* model, which could be explained by the production of sucrose-derived extracellular polysaccharides (EPS), increasing the bulk and porosity of the dental biofilm matrix [36].

This is the first *in situ* study which attempted to check a possible effect of a medically prescribed sugar-free pediatric antibiotic on the total microbiota counts. As a broad-spectrum antibiotic, amoxicillin/clavulanate potassium was expected to reduce total microbiota counts and enhances CFU counts of *Candida* spp when compared with its placebo [37]. These conditions were partially verified in the present study because although the antibiotic group showed higher CFU counts of *Candida* spp. in the presence of sucrose (especially at the high cariogenic challenge, *p*<0.05), lower total microbiota counts were not identified for the antibiotic when compared to the placebo at the 8-time daily frequency of sucrose exposure. Those contrasting results at the highest frequency of sucrose exposure should be interpret with caution because it could be due to an increased contribution of fungi in the total microbiota counts of the biofilm treated with amoxicillin/clavulanate potassium once blood agar plates were incubated in microaerophilic condition (candle jar).

In terms of microbial ecology, the DGGE technique has been a useful tool for detecting changes of predominant microbiota [38]. Concerning the oral microbiota, it has been successfully applied in the study of bacterial diversity of the oral microbiota in supragingival plaque from caries-free and caries-active individuals [38]–[40]; and in the investigation of the impact of anti-plaque agents like triclosan and chlorhexidine on the microbial ecology of *in vitro* dental plaque systems [41]–[42].

This is the first study in which the DGGE technique has been employed to evaluate possible changes in bacterial profile of a dental biofilm formed *in situ* and exposed to a medically prescribed antibiotic and its placebo. Irrespective of the frequency of sucrose exposure, the mean number of bands (number of bacterial species) detected in the DGGE profiles were higher for the placebo group when compared with antibiotic, but no statistically significant difference was found. Also, the mean rank of bands (mean position in the DGGE profile) was lower for placebo when compared with amoxicillin/clavulanate potassium, with no statistical significance between them for all the tested sucrose daily frequencies. The lack of statistical significance should be carefully interpreted because it could be due to the large inter-individual variability in the microbial profiles from the participants.

Cluster analyses of DGGE bacterial profiles from each volunteer demonstrated that the presence of sucrose in the *in situ* model increased, in some cases, the percentage similarity algorithm between samples of placebo and antibiotic when compared with the similarity observed between treatments' samples in the absence of sucrose. This finding could be due to a limitation of the DGGE technique characterized by the detection of nonviable microorganisms, which would be dependent on rapid turnover of dead cells and degradation of associated DNA within the evaluated microbial community [41]. This issue would, in part, explain why banding patterns were so similar for placebo and antibiotic in the presence of sucrose. In this situation, there would be more EPS in dental biofilm resulting in a thick undisturbed biomass, from which turnover of dead cells could hardly occur.

## Conclusion

Both conventional culture and DGGE have demonstrated some differences on total microbiota of the dental biofilm when exposed to antibiotic or placebo suspensions, mainly in the absence of sucrose. The lower total microbiota counts and total biofilm weight found in the antibiotic group at the 3-time daily frequency of sucrose exposure and in the absence of sucrose, could suggest a possible topical effect of the sugar-free amoxicillin/clavulanate potassium suspension on dental biofilm.

Further studies evaluating not only the topical effect of medically prescribed antibiotic suspensions on dental biofilm, but also on enamel hardness would contribute to clarify the fundamental issue of the protective effect of antibiotics on dental caries.
